# INTERGROWTH-21st *versus* a customized method for the prediction of neonatal nutritional status in hypertensive disorders of pregnancy

**DOI:** 10.1186/s12884-022-04450-3

**Published:** 2022-02-19

**Authors:** Juan Jesús Fernández-Alba, Maria Castillo Lara, Raquel Sánchez Mera, Sara Aragón Baizán, Carmen González Macías, Rocio Quintero Prado, Angel Vilar Sánchez, Jose Manuel Jimenez Heras, Luis Javier Moreno Corral, Francesc Figueras

**Affiliations:** 1 Department of Obstetrics and Gynaecology, University Hospital of Puerto Real, Puerto Real, Cadiz, Spain; 2 Institute of Research and Innovation in Biomedical Sciences of the Province of Cadiz (INiBICA). Fundación Cádiz - Hospital Universitario Puerto del Mar, 9º planta. Avda. Ana de Viya, 21 - 11009 Cadiz, Spain; 3 Nursing Department, Faculty of Nursing and Physiotherapy, Cadiz University, Cadiz, Spain; 4 Barcelona Center for Maternal–Fetal and Neonatal Medicine, Hospital Clínic and Hospital Sant Joan de Déu, IDIBAPS, University of Barcelona, Barcelona, Spain

**Keywords:** Foetal growth, Foetal malnutrition, Hypertensive disorders of pregnancy, Gestational hypertension, Small for gestational age (SGA)

## Abstract

**Background:**

Hypertensive disorders of pregnancy (HDP) generate complications and are one of the principal causes of maternal, foetal, and neonatal mortality worldwide. It has been observed that in pregnancies with HDP, the incidence of foetuses small for their gestational age (SGA) is twice as high as that in noncomplicated pregnancies. In women with HDP, the identification of foetuses (SGA) is substantially important, as management and follow-up are determined by this information.

**Objective:**

The objective of this study was to evaluate whether the INTERGROWTH-21st method or customized birthweight references better identify newborns with an abnormal nutritional status resulting from HDP.

**Method:**

A comparative analysis study was designed with two diagnostic methods for the prediction of neonatal nutritional status in pregnancies with HDP. The performance of both methods in identifying neonatal malnutrition (defined by a neonatal body mass index < 10^th^ centile or a ponderal index < 10^th^ centile) was assessed by calculating sensitivity, specificity, positive predictive value, negative predictive value, diagnostic odds ratio, Youden’s index and probability ratios.

**Results:**

The study included 226 pregnant women diagnosed with HDP. The customized method identified 45 foetuses as small for gestational age (19.9%), while the INTERGROWTH-21st method identified 27 newborns with SGA (11.9%). The difference between proportions was statistically significant (*p* < 0.01). Using body mass index (< 10^th^ centile) as a measure of nutritional status, newborns identified as SGA by the customized method showed a higher risk of malnutrition than those identified as SGA by INTERGROWTH-21st (RR: 4.87 (95% CI: 1.86–12.77) vs. 3.75 (95% CI: 1.49–9.43)) (DOR: 5.56 (95% CI: 1.82–16.98) vs. 4.84 (95% CI: 1.51–15.54)) Even when using Ponderal index (< 10^th^ centile), newborns identified as SGA by the customized method showed a higher risk of malnutrition than those identified as SGA by INTERGROWTH-21st (RR 2.37 (95% CI: 1.11–5.05) vs. 1.68 (95% CI: 0.70–4.03))(DOR 2.62 (95% CI: 1.00–6.87) vs. 1.90 (95% CI: 0.61–5.92)).

**Conclusion:**

In pregnant women with HDP, the predictive ability of the customized foetal growth curves to identify neonatal malnutrition appears to surpass that of INTERGROWTH-21st.

## Introduction

Hypertensive disorders of pregnancy (HDP), including gestational hypertension, chronic hypertension and preeclampsia (including superimposed preeclampsia), induce complications in approximately 10 to 16% of all pregnancies and are one of the principal causes of maternal, foetal, and neonatal mortality worldwide [1, 2]. The most frequent maternal complications include placental abruption, stroke and disseminated intravascular coagulation, while the associated foetal complications are intrauterine growth restriction, prematurity and intrauterine death [3–6]. The relationship between hypertension and alterations in foetal growth has been well demonstrated. In the context of HDP and secondary to placental involvement in the pathophysiology, there has been a reported and increased risk of small for gestational age (SGA) and foetal death [7, 8]. There is a biological gradient of risk across the different types of hypertension: gestational hypertension, mild preeclampsia and severe preeclampsia [9, 10]. Smallness for gestational age is a sentinel sign to detect babies in which growth restriction sources lay in placental dysfunction.

Traditionally, foetal growth has been evaluated by comparing estimated foetal weights with population-based reference curves. Likewise, some recent reports of the INTERGROWTH-21st project recommend using a single standard for both foetal growth and birthweight [11, 12].

Two competing approaches have been proposed for the detection of SGA, including the INTERGROWTH-21st project [11, 13] and a customized approach that assumes that one foetal growth standard does not fit all foetuses and has resulted in proposing customized standards [14, 15]. In the latter approach, the optimal weight at term for each fetus is estimated using a mathematical model estimated from foetal and maternal anthropometric variables [14, 15]. This predicted optimal weight at term can be combined with a foetal weight proportionality curve to calculate a customized curve for each mother in each pregnancy that can be used to predict ideal birthweight and foetal growth [13, 16–19].

We hypothesized that the customized method in pregnant women with hypertensive disorders would predict the nutritional status of newborns more accurately than INTERGROWTH-21st [19].

This study aimed to compare the performance of INTERGROWTH-21st methods vs. customized standards for the prediction of neonatal undernutrition in women with hypertensive disorders in pregnancy.

## Material and methods

### Design

This study was carried out as a retrospective historical cohort. The pregnancy data were retrieved from a single tertiary centre, the University Hospital of Puerto Real (Cádiz/Spain). Precisely, the data was retrieved from the clinical information system of the hospital. The study time range was comprised between January 2016 and March 2018. Only singleton births with HDP were included.

Newborns with malformations or congenital disorders or were stillborn were excluded from the study because of possible changes in foetal and birth weights. Gestational age was established based on the last menstruation and first ultrasound (usually at 11–12 weeks). In those cases where the gestational age deviated ≥ 1 week, the last menstruation was corrected and stored in the information system.

The study was approved by the local ethics committee: the Andalusia Biomedical Research Ethics Coordination Committee (CCEIBA) (Protocol number 0532-N-17).

### Definitions

A diagnosis of HDP was given when the patient presented a systolic blood pressure (BP) ≥ 140 mmHg and/or a diastolic BP ≥ 90 mmHg on two or more occasions spaced by 4–6 h [20]. In addition, HDP was classified as gestational hypertension, PEC without severe features, PEC with severe features, or chronic hypertension with superimposed PEC based on ACOG definitions [21]. Home surveillance was issued with diastolic BP values of 90 mmHg, and hospitalization was decided upon when proteinuria existed (defined by a 24-h collection) associated with a diastolic BP ≥ 100 mmHg [22, 23]. In all cases, an ultrasound examination was performed within two weeks before birth. In this ultrasound, foetal weights have been estimated using Hadlock's formula [24], and based on the estimated weights, the foetuses were classified as SGA (weight < 10^th^ centile), AGA (weight between 10 to 90^th^ percentile) or LGA (weight > 90^th^ centile) using both INTERGROWTH-21st and our own customized curves. A complete explanation of our customized method used in this study can be found in Fernández Alba et al. [25] This method (based on the proposal by Gardosi [26]) predicts the ideal weight that the newborn will have at 40 weeks as a function of its foetal sex and some maternal variables (age, height, and weight at the beginning of the pregnancy) using the following formula:

$$Birth Weight=1407.501+\left(maternal age x 4.087\right)+\left(pregestational weight x 6.506\right)+\left(maternal height x 8.716\right)+(newborn sex x 150.375)$$

where pregestational weight is calculated in kilograms, height in centimetres, and sex is codified as 1 if male and as 0 if female.

Then, the weight at each gestational age is calculated as a proportion of this estimated weight at 40 weeks, according to the proportionality curve proposed by Gardosi et al. [27], which is shown in the following formula:

$$\% foetal weight=299.1-\left(31.85 x GA\right)+\left(1.094 x {GA}^{2}\right)-0.01055 x ({GA}^{3})$$

where % foetal weight is the proportion of the foetal weight estimated at 40 weeks that corresponds to GA. For example, at 34 weeks of gestation, the foetal weight was 299.1 – (31.85 × 34) + (1094 × GA^2^) – 0.01055 X (GA) = 66.21%

That is, theoretically, a 34-week fetus weighs 66.21% of what it will weigh at 40 weeks of gestation. Based on the above, it is possible to plot a custom growth curve for each specific foetus.

The z score for a specific case can be calculated from the weight estimated by ultrasound, the ideal weight that corresponds to that GA and sex, and the coefficient of variation (0.12) using the following formula:

$$z=\left(\frac{weight estimated by ultrasound}{ideal weight}-1\right) \frac{1}{coefficient of variation}$$

The desired percentile will be obtained as the cumulative probability below this z score in a standard normal distribution.

Suspicion of SGA was established prenatally when the foetal weight, estimated with an ultrasound, was below the 10^th^ centile for gestational age and sex.

The birthweight was evaluated using a SECA (SECA, Hamburg, Germany) scale, with a precision of up to 100 g. The birth length of the newborns was taken using a model 210 SECA measuring mat/height rod with a 5-mm graduation measuring range.

Nutritional status was evaluated using two anthropometric methods: neonatal body mass index and ponderal index.

1. Neonatal body mass index (BMI): The BMI was calculated using the formula BMI = weight (kg) / height^2^ (m^2^) [28]. To assess nutritional status based on BMI, we used our own neonatal BMI table as a reference (Table 1). This reference table was created from our own database. The percentile of the BMI was calculated by multiplying the *p* value from the observed z score × 100. Newborns with a BMI between the 10^th^ centile and 90^th^ centile were classified as normal [29, 30]. Newborns with a BMI below the 10^th^ centile were classified as undernourished. Finally, newborns with BMI above the 90^th^ centile were classified as over nourished and thus were excluded from the analysis.
2. Neonatal ponderal index (PI): The PI was calculated using the formula PI = (weight in grams × 100) / (height in cm^3^) [31]. Rohrer's PI was adjusted for sex and gestational age [32]. To assess nutritional status based on PI, we used our own neonatal PI table as a reference (Table 2). This reference table was created from our own database [33]. The PI centile was calculated by multiplying the *p* value from the observed z score × 100. Newborns with PIs between the 10^th^ centile and 90^th^ centile were classified as normal. Newborns with PIs below the 10^th^ percentile were classified as undernourished. Finally, newborns with PI above the 90^th^ centile were classified as over nourished and, thus, were excluded from the analysis [34, 35].

**Table 1:** Neonatal body mass index adjusted for gestational age and sex

Gestational age (weeks)	Male		Female	
	**Mean**	**SD**	**Mean**	**SD**
**32**	10.44	1.18	10.25	0.74
**33**	10.66	1.17	10.19	1.02
**34**	10.59	1.08	10.77	1.42
**35**	11.90	1.45	11.84	2.14
**36**	12.39	2.07	12.08	1.50
**37**	12.60	1.36	12.59	1.59
**38**	13.25	1.36	13.18	1.60
**39**	13.41	1.17	13.30	1.25
**40**	13.67	1.47	13.58	1.26
**41**	13.84	1.45	13.82	1.30
**42**	14.12	1.23	13.72	1.34

^a^Based on our own population of 8928 newborns

*SD* Standard Deviation

**Table 2:** Neonatal ponderal index adjusted for gestational age and sex

Gestational age (weeks)	Male		Female	
	**Mean**	**SD**	**Mean**	**SD**
**32**	2.43	0.26	2.42	0.14
**33**	2.41	0.20	2.35	0.26
**34**	2.35	0.19	2.42	0.30
**35**	2.54	0.28	2.61	0.72
**36**	2.64	0.59	2.59	0.30
**37**	2.62	0.31	2.66	0.42
**38**	2.70	0.34	2.74	0.46
**39**	2.69	0.25	2.72	0.29
**40**	2.73	0.42	2.75	0.31
**41**	2.72	0.39	2.76	0.35
**42**	2.79	0.18	2.78	0.30

^a^Based on our own population of 8928 newborns

*SD* Standard Deviation

### Statistical analysis

Categorical data were summarized as counts and percentages. The distributions of continuous data were assessed using the Shapiro–Wilk test. Continuous data with a normal distribution are summarized as the mean and standard deviation. Conversely, when the data showed a nonnormal distribution, we used the median and the interquartile range as a measure of central tendency. The χ2 test was used to evaluate differences in the frequency of SGA and LGA newborns based on each classification method. The adjusted RRs obtained were compared to verify whether there were statistically significant differences [36].

To compare both methods of identification (INTERGROWTH-21st and customized), the following analyses were performed:

- Determination of the risk of alterations in the nutritional status of newborns (malnutrition). To calculate the risk of neonatal malnutrition in newborns, first, the exposed group was composed of those newborns classified as SGA, and the nonexposed group was composed of those classified as AGA. Later, the relative risk of malnutrition was calculated. The same analysis was carried out using both methods: INTERGROWTH-21st and our customized foetal growth curves. To check if there were significant differences, the risks obtained were compared using the method proposed by Altman and Bland [37].
- Sensitivity, specificity, positive (PPV) and negative (NPV) predictive values, positive and negative likelihood ratios (LR + and LR–), diagnostic odds ratio (DOR) and Youden’s index (sensitivity + specificity − 1) and their 95% CIs for each of the two methods were calculated to predict malnutrition of the newborns. Forest plots were produced. The diagnostic odds ratio (DOR) was defined as the ratio of the odds of the test being positive if the subject has a disease relative to the odds of the test being positive if the subject does not have the disease (which is also related to the likelihood ratios as LR + /LR-) [38]. The Youden index (sensitivity + specificity − 1) was calculated to determine the best compromise between sensitivity and specificity; the closer the value is to 1, the greater the diagnostic power [39]. To check if there were significant differences, the sensitivity and specificity of both methods were compared using the McNemar test [40].

For the statistical analysis of the data, we used the software R version 3.6.3 [41].

## Results

Our study included 226 pregnant women diagnosed with some form of HDP, including gestational hypertension (53.9%), chronic hypertension (26%) and preeclampsia (including superimposed preeclampsia) (20.1%). Table 4 shows the baseline characteristics of the included women. We found a mean maternal BMI (body mass index) of 29.9 ± 6.5 kg/m^2^ and a large proportion of pregnant women with obesity (45.9%) or overweight (27.6%) (Table 3).

**Table 3:** Maternal characteristics and perinatal outcomes

Variable	Value
**Maternal age (years):**	32.6 ± 5.3
**Maternal height (cm):**	162.02 ± 6.16
**Maternal BMI at the beginning of pregnancy (kg/m**^**2**^**):**	29.93 ± 6.52
-Underweight (BMI < 18.5)	14 (6. 2%)
-Normal BMI (18.5–24.9)	46 (20.3%)
-Overweight (BMI 25–29.9)	62 (27.6%)
-Obesity (BMI ≥ 30)	104 (45.9%)
**Hypertensive disorders of pregnancy**	
-Gestational hypertension	122 (53.9%)
-Chronic hypertension	59 (26%)
-Preeclampsia	45 (20.1%)
**Gestational age at the ultrasound scan (weeks):**	36.9 ± 2.4
**Estimated foetal weight (g)**	2789 ± 760
**Gestational age at birth (weeks):**	
Mean ± SD	37.7 ± 2.2
- < 34 weeks	16 (7.1%)
-34 – 34 + 6 weeks	31 (13.9%)
-37 – 40 + 6 weeks	1665 (73.2%)
- ≥ 41 weeks	13 (5.8%)
**Neonatal sex**	
-Female	115 (50.8%)
-Male	111 (49.2%)
**Birth weight (g)**	2978 ± 703
**Birth length (cm)**	48.33 ± 3.09
**Neonatal BMI at birth**	12.58 ± 2.02
**Malnourished newborns using BMI as reference**	19 (8.41%)
**Neonatal PI at birth**	2.61 ± 0.33
**Malnourished newborns using PI as reference**	26 (11.50%)
**Apgar score at 1 min**	
< 7	10 (4.40%)
≥ 7	216 (95.60%)
**Apgar score at 5 min**	
< 7	1 (0.27%)
≥ 7	225 (99.73%)

Data are given as n (%) or mean ± SD

*BMI* Body Mass Index, *PI* Ponderal Index

Using neonatal BMI as a reference, the incidence of newborns undernourished (BMI < 10^th^ centile) was 8.41%. When using neonatal PI as a reference, the incidence of newborns undernourished was 11.50%.

The incidence of SGA, AGA and LGA varied based on the reference method used. Using the customized method, we found 19.9% were SGA, 71.2% were AGA and 8.9% were LGA. However, using INTERGROWTH-21st, the incidence of SGA decreased to 11.9%, while the incidence of AGA was 71.2%, and the incidence of LGA was 14.2%. The differences between these proportions were statistically significant (*p* < 0.001) (Table 4).

**Table 4:** Classification of foetuses according to our customized method and INTERGROWTH21st

	**Customized method**	**INTERGROWTH21st**
	**N (%)**	**N (%)**
**SGA**	45 (19.9%)	27 (11.9%)
**LGA**	20 (8.8%)	32 (14.2%)
**AGA**	161 (71.2%)	167 (73.9%)

*SGA* Small for Gestational Age, *AGA* Adequate for Gestational Age, *LGA* Large for Gestational Age

The customized method identified 45 SGA newborns, while INTERGROWTH-21st identified 27 SGA newborns.

### Neonatal body mass index

**Fig. 1:**
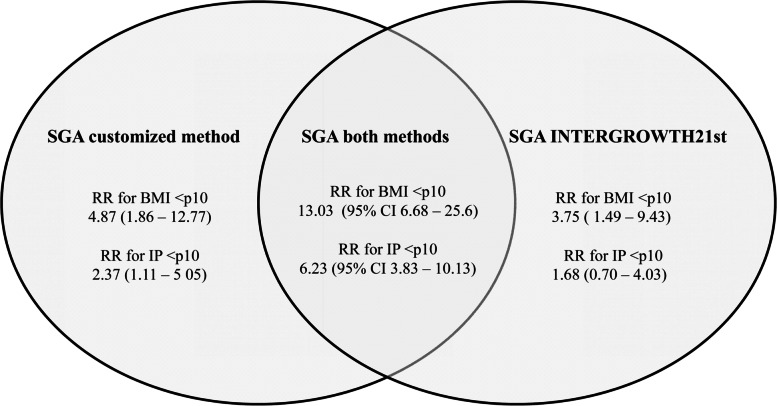
Venn diagram showing the RRs of being classified as SGA by the customized, INTERGROWTH-21st or both methods

**Fig. 2:**
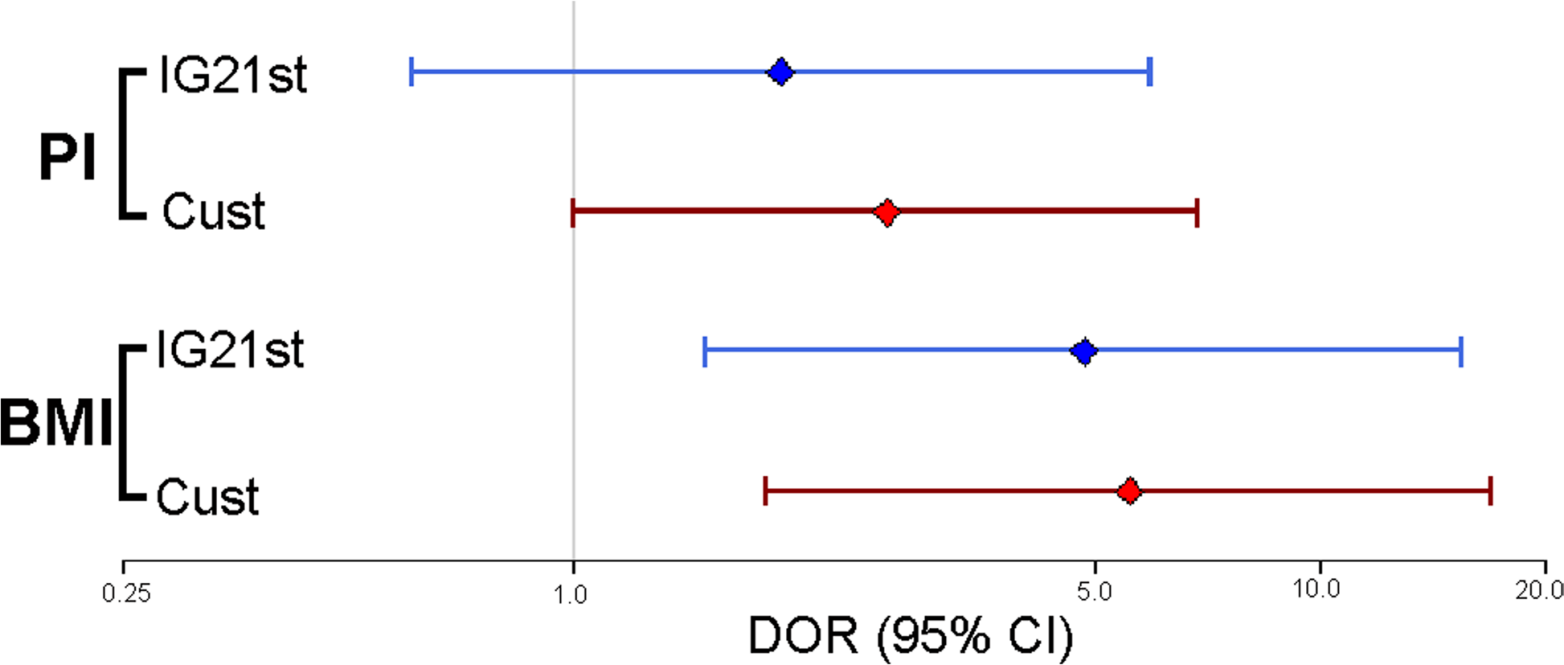
The diagnostic odds ratio (DOR) of INTERGROWTH-21st and the customized method in the prediction of neonatal malnutrition using PI and BMI < 10^th^ centile as references

In the entire population studied (term and preterm), using BMI (< 10^th^ centile) as a measure of nutritional status, the foetuses identified as SGA by the customized method seemed to show a higher risk of malnutrition than those identified as SGA by INTERGROWTH-21st (RR: 4.87 (95% CI: 1.86–12.77) vs. 3.75 (95% CI: 1.49–9.43)). Newborns classified as SGA by both methods showed an even higher risk of malnutrition (RR: 13.03; 95% CI: 6.68–25.60) (Fig. 1 and Table 5). The RRs were not significantly different between the two methods. The DOR was higher in the customized method (DOR: 5.56 (95% CI: 1.82–16.98) vs. 4.84 (95% CI: 1.51–15.54)), which suggests a higher discriminatory accuracy in the diagnosis of malnutrition (Fig. 2)*.*

**Table 5:** Relative risks of malnutrition in foetuses classified as SGA by INTERGROWTH21st and our customized method using PI (< 10^th^ centile) and BMI (< 10^th^ centile) as a measure of the nutritional status

Malnutrition (BMI < 10^th^ centile)		
	**RR**	95% CI
**Customized method SGA**	4.87	(1.86 – 12.77)
**INTERGROWTH21STSGA**	3.75	(1.49–9.43)
**Malnutrition (PI < 10**^**th**^** centile)**		
	**RR**	95% CI
**Customized method SGA**	2.37	(1.11–5.05)
**INTERGROWTH21STSGA**	1.68	(0.70–4.03)

*RR* Relative Risk, *CI* Confidence Interval, *SGA* Small for Gestational Age

In addition, the customized method was more sensitive than INTERGROWTH-21st (60.0% *vs.* 40.0%), although its specificity was lower (87.90% *vs.* 80.43%) (Table 6). The NPV was higher with the customized method than with INTERGROWTH-21st (94.87% *vs*. 92.37%). For detecting neonatal malnutrition, the customized method obtained a PPV of 25.0% compared to the 28.57% obtained by INTERGROWTH-21st. Detection of malnutrition by the INTERGROWTH-21st method resulted in LR + and LR − values of 3.31 and 0.68, respectively. The customized method seemed to be better for ruling out, with reported LR + and LR − values of 2.82 and 0.51, respectively (Table 6). McNemar’s test for BMI malnutrition showed a *p* value of 0.25 and was thus not statistically significant. The Youden’s index was 0.39 (95% CI: 0.03–0.69) with the customized method and 0.28 (95% CI: 0.03–0.61) with INTERGROWTH-21st, as shown in Table 6.

**Table 6:** Sensitivity, specificity, predictive values, Youden index, diagnostic odds ratio (DOR) and likelihood ratios of the INTERGROWTH21st and customized method for the identification of neonatal malnutrition using neonatal BMI and PI

**Malnutrition = neonatal BMI < 10**^**th**^** centile**								
	**All**		**Term (37–42 weeks)**			**Preterm (< 37 weeks)**		
	**INTERGROWTH21st** **SGA**	**Customized method** **SGA**	**INTERGROWTH21st** **SGA**	**Customized method** **SGA**		**INTERGROWTH21st** **SGA**		**Customized method** **SGA**
**Sensitivity**	40.00 (16.34 – 67.71)	60.00 (32.22 – 83.66)	23.08 (5.04 – 53.81)	30.77 (9.09 – 61.43)		50 (11.81 – 88.18)		83.33 (35.88 – 99.58)
**Specificity**	87.90 (80.83 – 93.07)	78.74 (70.59 – 85.50)	93.16 (86.97 – 97.00)	90.16 (83.45 – 94.81)		77.77 (60.84 – 89.88)		54.05 (36.92 – 99.58)
**PPV**	28.57 (11.28–52.17)	25.00 (12.12 – 42.20)	27.27 (6.02 – 60.97)	25.00 (7.27 – 52.38)		27.27 (27.34 – 78.07)		22.73 (7.82 – 45.37)
**NPV**	92.37 (86.01–96.45)	94.34 (88.09 – 97.89)	91.60 (85.09 – 95.90)	92.44 (86.13 – 96.48)		90.32 (74.25 – 97.96)		95.24 (76.18 – 99.88)
**LR + **	3.31 (1.51–7.22)	2.82 (1.66 – 4.80)	3.37 (1.02 – 11.17)	3.13 (1.78 – 8.30)		2.25 (0.82 – 6.15)		1.81 (1.10 – 2.99)
**LR-**	0.68 (0.45–1.04)	0.51 (0.27–0.95)	0.82 (0.61 – 1.12)	0.77 (0.53 – 1.11)		0.64 (0.28 – 1.46)		0.31 (0.05 – 1.89)
**Youden** **index**	0.28 (0.03 – 0.61)	0.39 (0.03 – 0.69)	0.16 (-0.08 – 0.51)	0.21 (-0.07 – 0.56)		0.27 (-0.27 – 0.78)		0.37 (-0.27 – 0.70)
**DOR**	4.84 (1.51 – 15.54)	5.56 (1.82 – 16.98)	4.09 (0.93 – 17.89)	4.07 (1.09 – 15.25)		3.50 (0.59 – 20.81)		5.88 (0.62 – 55.38)
**Malnutrition = neonatal PI < 10**^**th**^** centile**								
	**All**		**Term**			**Preterm**		
	**INTERGROWTH21st** **SGA**	**Customized method** **SGA**	**INTERGROWTH21st** **SGA**		**Customized method** **SGA**	**INTERGROWTH21st** **SGA**	**Customized method** **SGA**	
**Sensitivity**	22.73 (7.82 – 45.37)	40.91 (20.71 – 63.64)	17.65 (3.80 – 43.43)		23.53 (6.81 – 49.90)	22.22 (2.81 – 60.00)	55.55 (21.20 – 86.30)	
**Specificity**	86.61 (78.87 – 92.31)	79.13 (70.56 – 86.15)	93.40 (86.87 – 97.30)		91.89 (85.16 – 96.22)	72.72 (54.47 – 86.70)	50.00 (32.42 – 67.57)	
**PPV**	25.00 (8.66 – 49.10)	27.27 (13.30 – 45.52)	30.00 (6.67 – 65.24)		30.77 (9.09 – 61.43)	18.18 (2.28 – 51.77)	22.73 (7.82 – 45.37)	
**NPV**	85.09 (77.20—91.07)	87.50 (79.57–93.17)	87.62 (80.09 – 93.06)		88.69 (81.44 – 93.84)	77.42 (58.90 – 90.40)	80.95 (58.09 – 94.55)	
**LR + **	1.70 (0.69–4.19)	1.96 (1.06 – 3.63)	2.67 (0.76 – 9.34)		2.90 (1.00 – 8.39)	0.81 (0.21–3.12)	1.11 (0.57 – 2.18)	
**LR-**	0.89 (0.70–1.13)	0.75 (0.52 – 1.07)	0.88 (0.70 – 1.10)		0.83 (0.63 – 1.09)	1.07 (0.71 – 1.60)	0.89 (0.40 – 1.99)	
**Youden** **index**	0.09 (-0.13 – 0.38)	0.20 (-0.09 – 0.50)	0.11 (-0.09 – 0.41)		0.15 (-0.08 – 0.46)	-0.05 (-0.43 – 0.47)	0.05 (-0.46 – 0.54)	
**DOR**	1.90 (0.61 – 5.92)	2.63 (1.00 – 6.87)	3.03 (0.70 – 13.10)		3,49 (0.94 – 12.94)	0.76 (0.13 – 4.37)	1.25 (0.28 – 5.47)	

*BMI* Body Mass Index, *SGA* Small for Gestational Age, *PPV* Positive Predictive Value, *NPV* Negative Predictive Value; *PI* Ponderal Index, *LR +*  Positive Likelihood Ratio, *LR-* Negative Likelihood Ratio, *DOR* Diagnostic Odds Ratio

It is important to note that in the same sample, the customized method identified 9 malnourished newborns, whereas INTERGROWTH-21st identified only 6 malnourished newborns.

Focusing on preterm newborns and using BMI as a reference, the sensitivity of the customized method was 83.33% (35.88–99.58) versus 50% (11.81–88.18) using INTERGROWTH-21st.

### Neonatal ponderal index

In the entire population studied (term and preterm), using PI (< 10^th^ centile) as a measure of nutritional status, the newborns identified as SGA by the customized method seemed to show a higher risk of malnutrition than those identified as SGA by INTERGROWTH-21st (RR: 2.37 (95% CI: 1.11–5.05) *vs.* 1.68 (95% CI: 0.70–4.03). On the other hand, newborns classified as SGA by both methods showed an even higher risk of malnutrition (RR: 6.23; 95% CI: 3.83–10.13) (Fig. 1 and Table 5). The results suggested that DOR was higher with the customized method (DOR: 2.62 (95% CI: 1.00–6.87) *vs.* 1.90 (95% CI: 0.61–5.92)) (Fig. 2).

The customized method seemed to be more sensitive than INTERGROWTH-21st (40.91% *vs.* 22.73%), although its specificity was lower (79.13% *vs.* 86.60%) (Table 6). Regarding the detection of neonatal malnutrition, the NPV was higher with the customized method (87.50%) than with INTERGROWTH-21st (85.09%). McNemar’s test for PI malnutrition showed a *p* value of 0.13 and was thus not statistically significant. The Youden indices were 0.20 and 0.09, respectively, as shown in Table 6. It is important to note that in the same sample, the customized method identified 9 undernourished newborns, whereas INTERGROWTH-21st identified only 5 undernourished newborns.

In preterm newborns and using the PI as a reference, the sensitivity of the customized method also increased (50%; 95% CI: 21.20–86.30). However, the sensitivity of INTERGROWTH-21st decreased to 22.22% (2.81–60.00) (Table 6).

### Comment

#### Principal findings

We have observed in newborns of mothers with HDP, different rates of SGA and LGA based on the reference curve used, namely, the INTERGROWTH-21st or customized curves. On one hand, the SGA rate using INTERGROWTH-21st was 11.9%, which was significantly lower than the 19.9% observed using customized curves. On the other hand, the LGA rate using INTERGROWTH-21st was 14.2%, compared to 8.9% using our customized curves as the reference. Consequently, in our population, the customized method identified more SGA while INTERGROWTH-21st identified more LGA.

On the other hand, our study suggested that in pregnant women with HDP, the accuracy of the customized method exceeded that of INTERGROWTH-21st in the identification of newborns with malnutrition. In the entire sample studied, the sensitivity of the customized method exceeded that of INTERGROWTH (60% vs. 40% based on BMI and 40.91% vs. 22.73% based on PI). This difference was even greater in the group of preterm newborns in which, using BMI as a reference, the sensitivity of the customized method was 83.33% compared to the 50% shown by INTERGROWTH-21st, and using PI, the sensitivity of the customized method was double that of INTERGROWTH-21st (55.55% vs. 22.22%).

Finally, using PI as a reference, the DOR of the customized method was higher than that of INTERGROWTH-21st in all the groups studied. Similarly, using BMI as a reference, the DOR of the customized method was higher both in the whole sample (5.56 *vs.* 4.84) and in the group of preterm newborns (5.88 *vs.* 3.50), and it was higher for INTERGROWTH-21st in the group of term newborns but with a minimal difference (4.09 *vs.* 4.07).

A correct classification of the foetal nutritional status using an appropriate model is fundamental to properly manage the pregnancy. A fetus incorrectly classified as SGA in any pregnancy, especially in those with HDP, who are at particular risk, will cause the obstetrician to increase the number of visits and the monitoring of the pregnant woman with HDP, as well as potentially lead to an unwarranted preterm or early term birth. Thus, it is crucial to select the method that best identifies newborns with real nutritional disorders (malnutrition). The lack of prenatal detection of malnutrition (i.e., false negatives) puts the baby at unnecessary risk by preventing them from close follow-up and timely delivery.

Our study showed the importance of adequately choosing the reference curve from which to carry out a screening or even diagnose a prenatal SGA foetus, especially with preterm foetuses. Since its sensitivity is higher, the customized method seems to be better for ruling in/identifying foetuses at higher risk that need intensive follow-up during pregnancy.

#### Comparison with existing literature

Other authors have found that the incidences of SGA, AGA and LGA were different when INTERGROWTH-21st was used compared to a customized method. A recent article published by our group found similar findings in pregnant women with gestational diabetes [42]. Our observations are supported by other publications, such as that of Fay et al. [16], who, in an unselected population of 125,826 pregnant women, found a proportion of SGA using the INTERGROWTH-21st method of only 4.5%, compared to the 10.9% that was found using their own customized method (GROW) [19]. Similarly, Anderson et al. [43], after comparing both methods in an unselected population of 53,484 pregnant women in New Zealand, reported proportions of SGA significantly lower using INTERGROWTH-21st than customized curves (4.5% *vs.* 11.6%). Francis et al. [17] obtained similar results in uncomplicated pregnancies, with a rate of SGA using customized curves of 10.5%, whereas the proportion of SGA based on INTERGROWTH-21st was 4.4%. In our study, the incidence of SGA was higher than that published by these authors. The higher proportion of SGA found in our study can be explained because we included only pregnant women with HDP, while most of the reviewed studies were carried out in low-risk populations.

Nonetheless, our results are comparable to the data observed by Allen et al. [7] who, using population curves in a Canadian population of 135,466 patients, described a proportion of SGA of 9.8% in uncomplicated pregnancies and 15.3% in pregnancies with HDP. Another study, which was also conducted in Canada, used customized curves, included 300,000 pregnant women, and reported incidences of SGA of 6.4% in uncomplicated pregnancies and 9.9%, 11.5% and 15.6% in patients with gestational hypertension, chronic hypertension and eclampsia, respectively [44].

A prior study carried out by our team in 2016 [35], in an unselected population, showed the superiority of the customized method over our own population-based method for the identification of newborns with a PI at birth < 10^th^ centile. This was more evident in the highest scales of maternal weight and height.

Similarly, in a recent study carried out by our team and focused on pregnant women with gestational diabetes, we compared our custom method against INTERGROWTH-21st for the identification of newborns with a PI > 90^th^ centile. In this study, our customized method showed a positive likelihood ratio higher than INTERGROWTH-21st (5.40 vs. 2.54).

Owen et al. [45] found a similar relationship between customized birth weight percentiles and neonatal malnutrition but concluded that in a low-risk population, the customized curves were only moderately useful in the identification of neonates with a low PI, with a positive likelihood ratio of 4.3 (95% CI: 2.5–7.1). Agarwal et al. [46] also found that the PI at birth was lower in newborns classified as SGA by customized curves than based on population-based curves.

#### Clinical and research implications

In this study, we found that using INTERGROWTH-21st, the incidence of SGA was lower than that found using customized curves (11.9% vs. 19.9%). Thus, in pregnant women with HDP, the customized method identified a significantly larger number of foetal SGA newborns than INTERGROWTH-21st. Given that INTERGROWTH-21st was built from a multiethnic population, its use in our population could be biased.

When comparing the two methods, we observed that both have a percentage (although small) of false negatives, although in our study, the diagnostic yield by the customized curve was shown to be superior.

In addition, 18 foetuses were classified as SGA by the customized method and as AGA by INTERGROWTH-21st. It is worth noting that in this group of foetuses, 22.22% (using PI as a reference) and 16.66% (using BMI) were malnourished.

Therefore, in the whole sample studied (term and preterm), a relevant number of cases of malnutrition in newborns were not detected by INTERGROWTH-21st. Regarding false-positives, we observed that foetuses identified as small only by the INTERGROWTH-21st standards did not have an increased risk of undernutrition at birth and, therefore, could be assumed to be constitutionally small.

Foetuses born with malnutrition who were prenatally misclassified (i.e., were false negatives with INTERGROWTH-21st or the customized method) could be particularly vulnerable and at higher risk of adverse perinatal outcomes. Therefore, it is important to accurately diagnose them to design a better medical approach and adequate health care.

Owing to the need to rule in the foetuses most likely at risk of malnutrition, we consider that a diagnostic method with a higher value of sensitivity will perform better in the clinic. Taking this into consideration, since INTERGROWTH-21st has proven to perform worse with regard to ruling-in than the customized method and those ruled-in by INTERGROWTH seem to be constitutionally small, we consider that our customized method can be superior to INTERGROWTH-21st in estimating the risk of malnutrition in pregnant women with HDP.

This is particularly important in the group of preterm newborns. In these children, the sensitivity of the custom curves was even higher (83.33 using BMI as the reference and 55.55 using PI as the reference). Population curves (including INTERGROWTH-21st) are constructed from the actual weights obtained at birth. In preterm infants, the use of birthweights introduces a bias in the curve since most premature babies are born due to pathological processes (iatrogenic, spontaneous preterm delivery, or placental-related diseases such as HDP), and for this reason, their weights should not be considered normal. The normative charts based on foetuses from normal pregnancies are referred to as foetal growth standards, while the descriptive foetal growth charts based on foetuses/infants from normal and complicated pregnancies are called references [47]. Understanding the difference between foetal growth standards and foetal growth references is critical for percentile-based normative interpretations. The use of a population-based percentile curve in preterm babies is likely to classify children who are truly small for gestational age as normal. However, the custom curve projects the foetal growth curve for all gestational ages as a ratio of the predicted ideal weight at 40 weeks, according to a normal foetal growth curve. This is most likely the reason why the customized method can identify more newborns that are truly small for their gestational age.

In our opinion, this indicates that the same cutoff point (10^th^ centile for SGA) can result in a child being classified as normal or small depending on the reference curve. This is especially important in the follow-up of pregnant women with HDP in whom the risk of intrauterine growth restriction is particularly high.

#### Strengths of the study and limitations

Our study was not exempt from limitations. Although it is more widely used, the PI is not a true reflection of nutritional status, but it is useful for comparing the nutritional status of newborns in similar studies. In children, BMI has become a good parameter to determine nutritional status, corporal proportions and adiposity [43, 48]. Nonetheless, the BMI values during the prenatal period have not yet been studied appropriately. They have been proposed as a useful parameter for the classification of newborns with states of nutritional alterations, contributing to better detection of intrauterine growth disorders [49].

Even though anthropometric index presents some limitations to evaluate nutritional status in clinical practice, their utility in epidemiological studies has been accepted. Although there are many indices composed of weight and length, the BMI and PI seems to be the most accurate index to evaluate fat mass in newborns.

A recent study published by Chen et al. [32] informs us that although skinfold measures may have more discriminative power in terms of total body adiposity, simple anthropometric measures (such as PI or BMI) correlated strongly with neonatal adiposity and concluded that these simple measures could be of value in epidemiological studies.

Moreover, a recent article comparing BMI with PI and the weight-for-length ratio in preterm infants [50] concluded that BMI appeared to be the best single measure of body proportionality in preterm babies, which contrasts with current practice.

On the other hand, there has been no proper validation of INTERGROWTH-21st for its use in our population. However, our own custom curve underwent a cross-validation process.

Another limitation is related to the sample size. A larger sample size might result in narrower CIs with statistically significant differences between the groups. Moreover, a larger sample size might provide a decreased *p* value in the McNemar test, making the customized method more adequate than the INTERGROWTH-21st method.

## Conclusions

In pregnant women with HDP, the capacity of the customized growth curves to identify newborns with undernutrition seems to exceed that of INTERGROWTH-21st. However, further studies with a larger sample size of patients are necessary to confirm these findings.

## Data Availability

The data that support the findings of this study are available from the corresponding author (Castillo M.) upon reasonable request.

## Abbreviations

AGA: Adequate for gestational age
BMI: Body mass index
CI: Confidence interval
DOR: Diagnostic odds ratio
EFW: Estimated foetal weight
HDP: Hypertensive disorders in pregnancy
LGA: Large for gestational age
LR-: Negative likelihood ratio
LR +: Positive likelihood ratio
NPV: Negative predictive value
PI: Ponderal index
PPV: Positive predictive value
RR: Relative risk
SGA: Small for gestational age
